# A Case of a Pigmented Epithelioid Melanocytoma on a Mucosal Site

**DOI:** 10.5826/dpc.1004a70

**Published:** 2020-10-26

**Authors:** Alice Ramondetta, Simone Ribero, Luca Conti, Pietro Quaglino, Paolo Broganelli

**Affiliations:** 1 Dermatology Clinic and Surgical Pathology Section, Department of Medical Sciences, University of Turin, Italy

**Keywords:** melanocytoma, epithelioid cells, vulva, genital dermatopathology, melanocytic lesion

## Introduction

Pigmented epithelioid melanocytoma (PEM) is an uncommon and recently described entity with unknown biologic behavior. It is a melanocytic tumor showing overlapping features of both an atypical epithelioid blue nevus and a low-grade “animal-type melanoma.”

PEM occurs over a broad age range, with a predilection for children and young adults, and clinically appears as a macular, popular, or nodular lesion. Dermoscopically, PEM appears as a polymorphic lesion, characterized by homogeneous blue pigmentation and a combination of black, brown, and white colors. Histopathologically, it is characterized by a dermal proliferation of heavily pigmented, both dendritic and spindle/epithelioid melanocytes, admixed with slightly larger, plumper, and less pigmented epithelioid cells [1]. Involvement of the regional nodes has been reported but usually with no further spread of the disease. No histological criteria are predictive of metastatic behavior.

## Case Presentation

**Figure 1:**
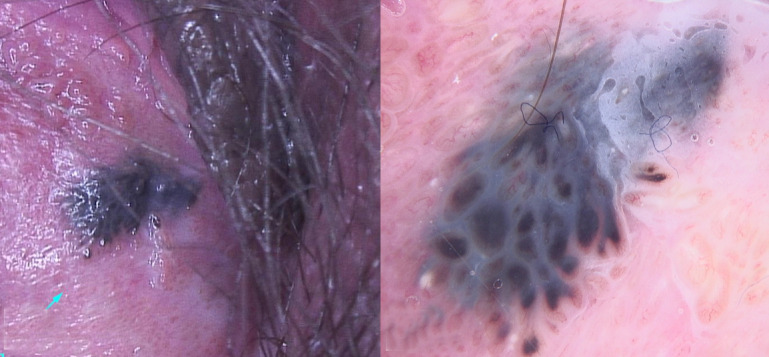
(A) Clinical appearance: oval papule, brown-black in color, intensely pigmented, about 1 cm in size. (B) Dermoscopy: many irregular, black blotches surrounded by a whitish blue veil. No atypical vascular feature was detected.

We describe a case of vulvar mucosal PEM, that occurred on the right small lip of a 50-year-old woman. Clinically it appeared as an intensely pigmented, brown-black papule about 1 cm in size. On dermoscopy, we observed many irregular, black blotches surrounded by a whitish blue veil. No atypical vascular feature was detected. Due to its globular structure and black-bluish color and considering the age of the patient and rapid onset, the lesion resembled a Spitz nevus or an atypical Spitz tumor (Figure 1, A and B). However, spitzoid lesions do not show these dermoscopic features. Therefore, an excisional biopsy was performed.

**Figure 2:**
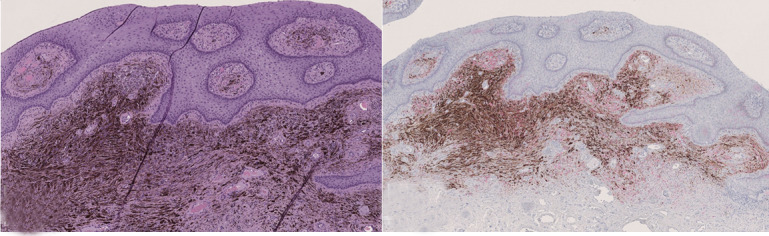
(A) Histopathological specimen: acanthotic epidermis with a slightly warty profile overlying a large dermal nodular lesion with blurred limits consisting of hyperpigmented epithelioid and spindle dendritic melanocytes arranged in perivascular and periadnexal bundles, mixed with epithelioid nevoid cells and numerous melanophages. No significant atypia, mitosis, or necrosis was appreciated. Immunohistochemical staining: HMB-45, Melan-A (B) and p16 (all expressed), ki-67 (1%).

The histological examination showed an acanthotic epidermis with a slightly warty profile overlying a large dermal nodular lesion with blurred borders consisting of hyperpigmented epithelioid and spindle dendritic melanocytes arranged in perivascular and periadnexal bundles, mixed with epithelioid nevoid cells and numerous melanophages. No significant atypia, mitosis, or necrosis was appreciated. Immunohistochemical staining was performed. While HMB-45 and Melan-A were positive, p16 was expressed on the entire lesion, and KI-67 showed low activity (1%) (Figure 2, A and B).

## Conclusions

Pigmented lesions of mucosal sites usually can be difficult to interpret. PEM is a rare entity, and we report on a case of a female genital mucosal variant. Uehara et al published a case of PEM on the glans [2]. PEM could mimic both benign and malignant lesions, from which it must be distinguished, albeit with difficulty.
